# Volume EM: A quiet revolution takes shape

**DOI:** 10.1038/s41592-023-01861-8

**Published:** 2023-04-19

**Authors:** Lucy M. Collinson, Carles Bosch, Anwen Bullen, Jemima J. Burden, Raffaella Carzaniga, Cheng Cheng, Michele C. Darrow, Georgina Fletcher, Errin Johnson, Kedar Narayan, Christopher J. Peddie, Martyn Winn, Charles Wood, Ardan Patwardhan, Gerard J. Kleywegt, Paul Verkade

**Affiliations:** 1 Electron Microscopy Science Technology Platform, Francis Crick Institute, 1 Midland Road, London NW1 1AT, UK; 2 Sensory Circuits and Neurotechnology Laboratory, Francis Crick Institute, 1 Midland Road, London NW1 1AT, UK; 3 UCL Ear Institute, University College London, 332 Gray’s Inn Road, London WC1X 8EE, UK; 4 Laboratory for Molecular Cell Biology, University College London, Gower Street, London, WC1E 6BT, UK; 5 Zeiss Research Microscopy Solutions, Carl Zeiss Ltd, Zeiss Group, Cambourne, United Kingdom; 6 ConnectomX Ltd, Sweetmans Cottage, Yarnells Hill, Oxford, OX2 9BG, UK; 7 Artificial Intelligence & Informatics, The Rosalind Franklin Institute, Harwell Science Campus, Didcot, UK OX11 0QS; SPT Labtech Ltd., Melbourn, UK SG8 6HB; 8 BioImagingUK, 37/38 St Clements, Oxford, OX4 1AJ, UK; 9 Dunn School Bioimaging Facility, Sir William Dunn School of Pathology, Oxford University, Oxford, OX1 3RE, UK; 10 Center for Molecular Microscopy, Center for Cancer Research, National Cancer Institute, National Institutes of Health, Bethesda, Maryland, USA, & Cancer Research Technology Program, Frederick National Laboratory for Cancer Research, Frederick, Maryland, USA; 11 UKRI-STFC, Rutherford Appleton Laboratory, Research Complex at Harwell, Didcot OX11 0FA, United Kingdom; 12 Future Technology Centre, School of Mechanical and Design Engineering, University of Portsmouth, Portland Building, Portland Street, Portsmouth, PO1 3HE; 13 European Molecular Biology Laboratory, European Bioinformatics Institute (EMBL-EBI), Wellcome Genome Campus, Hinxton, Cambridge CB10 1SD, UK; 14 School of Biochemistry, University of Bristol, University Walk, Bristol, BS8 1TD, UK

## Abstract

Volume Electron Microscopy is a group of techniques that reveal the 3D ultrastructure of cells and tissues through volumes greater than 1 cubic micron. A burgeoning grass roots community effort is fast building the profile, and revealing the impact, of vEM technology in the life sciences and clinical research.

## Imaging as a key technology

The award of three Nobel Prizes for imaging technologies between 2008 and 2017 has highlighted the key importance of imaging in present day life science research. The expression of proteins tagged with Green Fluorescent Protein (GFP) in living cells and organisms transformed the way life science research was conducted. The ability to visualise the dynamic nature of proteins in cells and tissues was recognised with the Nobel Prize in Chemistry in 2008. This was followed by another Nobel Prize in Chemistry in 2014 for the development of super-resolution light microscopy technologies, in which Abbe’s resolution limit was finally broken, allowing localisation of fluorescently tagged molecules with a precision of tens of nanometres. Most recently, the ‘resolution revolution’ in cryogenic electron microscopy (cryo-EM) has enabled determination of the molecular structure of isolated proteins and protein complexes and was recognised with the 2017 Nobel Prize in Chemistry. There is now another imaging revolution underway that reveals the exquisite complexity of cells and tissues at the membrane and organelle scale in three dimensions, volume Electron Microscopy (vEM), which was recently highlighted as one of the “Seven Technologies to Watch in 2023” by Nature (https://www.nature.com/articles/d41586-023-00178-y).

## What is vEM?

**Figure 1:**
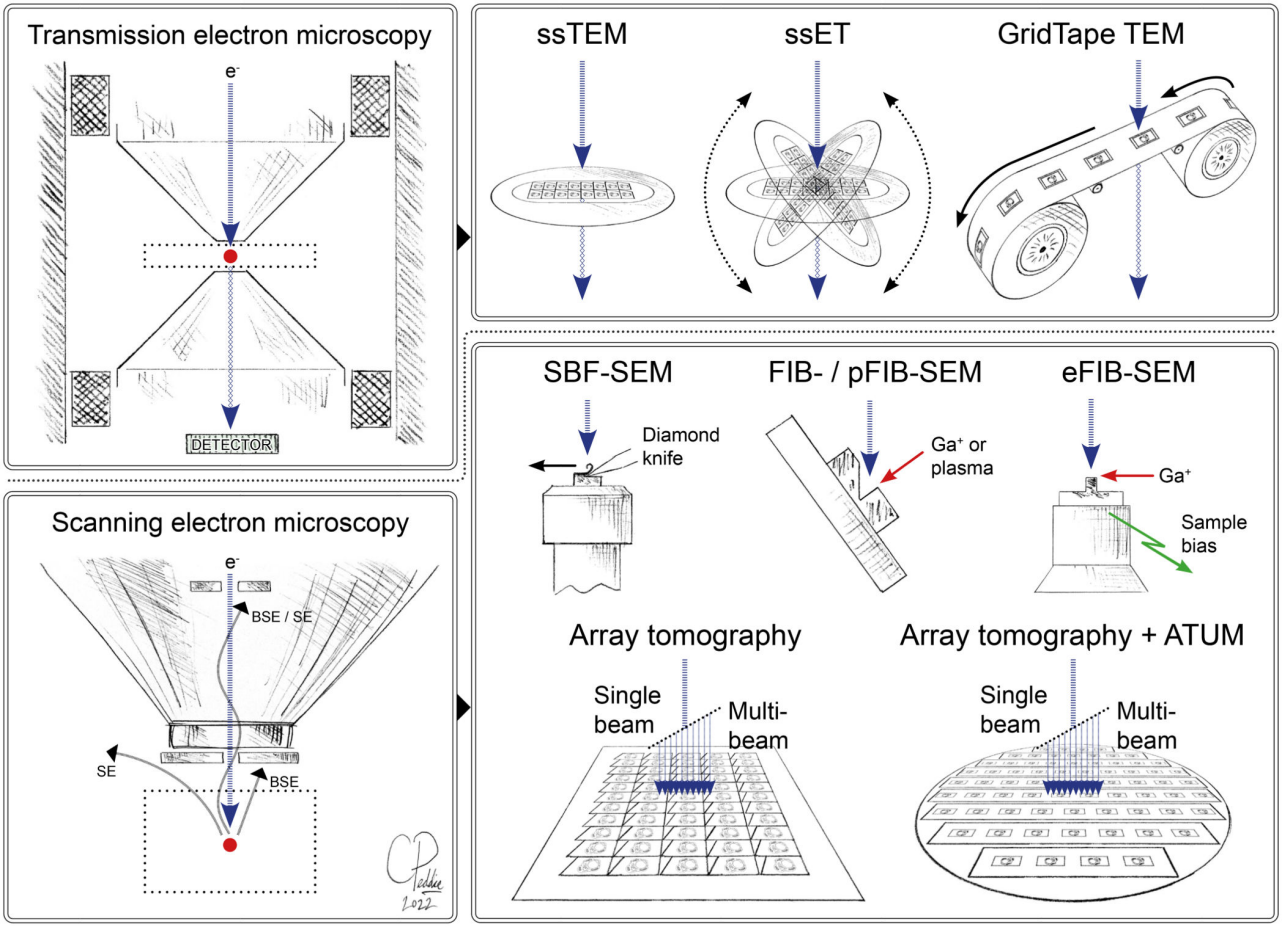
Volume EM technologies. vEM encompasses a range of technologies based on transmission and scanning electron microscopes, that enable collection of a series of images through the volume of resin-embedded cells and tissues ^1^. The source (e^-^) generates a beam of electrons (blue arrows) which passes through (in the TEM) or interacts with (in the SEM) the sample (red dot) and forms an image on the detector. The image is generated by scattering of the primary electron beam in the TEM, and by secondary electrons or backscattered electrons in the SEM. TEM (top row) can be used for volume EM by sequential imaging of serial sections on grids (ssTEM serial section transmission electron microscopy; ssET – serial section electron tomography) or on tape (GridTape TEM). SEM (bottom row) can be used for vEM by sequential imaging of an exposed sample surface cut with a diamond knife (SBF-SEM – serial blockface scanning electron microscopy) or ion beam (FIB-SEM – focused ion beam scanning electron microscopy; pFIB-SEM – plasma FIB-SEM; eFIB-SEM – enhanced FIB-SEM), or by sequential imaging of serial sections on a substrate such as a silicon wafer (array tomography). Ultramicrotomy may be partially automated by collecting sections onto tape (ATUM – automated tape ultramicrotomy). Ga^+^ - gallium ions. Original artwork by Chris Peddie @TheCrick.

vEM is a group of techniques used to image the structure of cells and tissues through continuous volumes of at least one cubic micron at nanometer resolution ^1^ (Fig.1). vEM encompasses imaging technologies based on both transmission electron microscopy (TEM) and scanning electron microscopy (SEM). In all cases, vEM generates a series of images of the specimen that, when combined, form a digital representation of the specimen volume. The individual images are acquired by (repeatedly) slicing the specimen into thin sections, and then imaging either the sections (from different angles in the case of tomography) or the exposed face of the sample. Without using the exact term, various kinds of vEM have been in use for decades, contributing significantly to our understanding of the complexity of cells, tissues, and organisms.

## What can vEM reveal about life?

**Figure 2:**
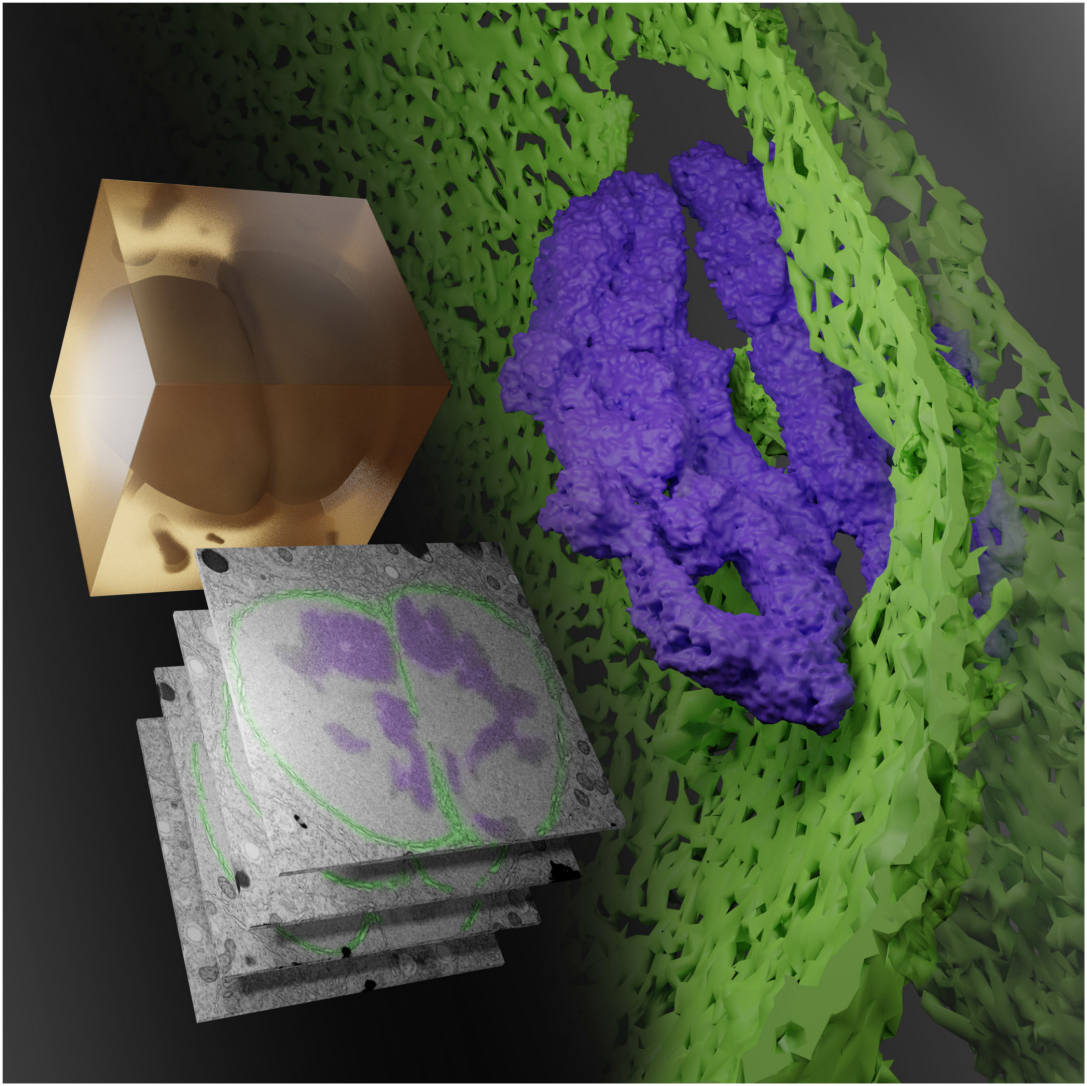
First contact. Shown is a rendering of a volume EM (vEM) workflow to capture in 3D and at high resolution the coming together of haploid parental genomes following fertilization. A high-pressure frozen, stained and resin-embedded *C.elegans* zygote (amber block) was imaged by FIB-SEM to generate a stack of grayscale images of the targeted volume. Features of interest were reconstructed in 3D, revealing the fenestrations in the breaking down nuclear membrane (false coloured green in 3D and highlighted in green in grayscale images) through which the condensed parental genomes (purple) make contact before full mixing. Image credit: Joseph Meyer, Kedar Narayan ^7^.

vEM reveals the structural beauty and complexity of life, from the membranes that make up organelles, to the arrangement of organelles within cells, to the communities of cells that make up tissues, and the architecture of tissues that make up organisms. This makes vEM a critical tool for understanding biological complexity across scales. Indeed, the development of vEM was originally driven by the quest to understand the connections in the brain, from individual vesicles releasing neurotransmitters at synapses to entire neurons making connections across different brain regions. Since the 1980s, vEM has delivered connectomes from model organisms including *C.elegans* ^2^, *D.melanogaster* ^3–5^ and *D.rerio* 6. However, complexity across scales is present in every organism, and vEM is now being used throughout the life sciences, revealing the structural complexity of fertilization ^7^ (Fig.2), blood vessels ^8,9^, muscles ^10^, sensory organs ^11,12^, tumours ^13,14^, pathogen-infected cells and tissues ^15,16^, plants ^17,18^ and marine organisms ^19,20^, to name but a few.

## vEM workflows

There are three main components common to all vEM workflows: 1) Sample preparation, 2) Imaging, and 3) Data.

In the first step, the sample is prepared by chemical or cryogenic fixation, followed by staining using heavy metal salts of osmium, lead and uranium to add electron contrast to the membranes and make the sample more conductive. The sample is then dehydrated using a solvent, infiltrated with a liquid resin, and the resin polymerised using heat or UV light. This encases the cells and tissues like a mosquito in amber, resulting in a hard block that can be sliced using a diamond knife or an ion beam. Slicing is an essential part of the vEM workflow because of the poor penetration of the electron beam into the samples. Slicing, or sectioning, may be performed manually on an ultramicrotome using a diamond knife, and the ultrathin sections collected onto metal grids, tape, or wafers for imaging. Alternatively, the sections may be removed and discarded, and the block surface imaged after each cut. In both cases, the result is a set of sequential images that represent the volume of the original sample.

In the second step, vEM imaging is performed using TEM or SEM (Fig.1). TEM methods include imaging of serial sections on grids (ssTEM), and serial section electron tomography (ssET), which improves axial resolution. SEM-based vEM techniques became viable following the switch from analog to digital electronics and improvements in sources and detectors, resulting in TEM-like imaging in the SEM. Serial Block Face SEM (SBF-SEM) incorporates an entire ultramicrotome device within the SEM chamber, allowing some level of automation of the slicing and imaging cycle ^21^. Focused Ion Beam SEM (FIB-SEM) adds a second column to the chamber, using gallium ions to mill away thin layers of the specimen surface prior to imaging the blockface ^22^. Array tomography uses large silicon or conductive glass substrates to hold hundreds or thousands of ultrathin sections for serial imaging in an array format ^23^. We refer to the recent review by Peddie et al. ^1^ for further details of the different vEM techniques.

In the third step, the vEM data is processed and analysed. vEM now joins light sheet microscopy and cryo-EM in catapulting the life sciences into the ‘big image data generator’ regime and approaching data output levels more familiar in astrophysics and particle physics. A single vEM instrument can easily generate 250 GB of image data per day, with new high-speed imaging systems promising rates of 5+ TB per hour. In addition, considering that many vEM experiments involve correlative and multimodal workflows, the complexity of the data is multiplied. This gives rise to heterogeneity in data types, file formats and branched data flows within a single experiment, requiring the development of a coherent global data model. Much of the data generated by vEM workflows thus remains un-analysed due to the rich content of the images and the lack of automated image analysis methods available, though this is changing quickly with the explosion of artificial intelligence methods. Public archiving of data and user-friendly implementations of advanced analysis algorithms will be critical to ensure that vEM data is fully mined. The EMPIAR archive ^24^ (https://empiar.org/), although originally developed to host raw cryo-EM datasets, now supports archiving of vEM data as well (currently ~10% of new depositions). EMPIAR is working actively with the community to further accommodate the requirements of vEM data producers as well as users from communities as diverse as cell biology and machine learning. The connectomics community have developed their own solutions for sharing vEM data, including the Open Connectome Project (https://neurodata.io/project/ocp/), the Registry of Open Data on AWS (Open NeuroData; https://registry.opendata.aws/open-neurodata/), and 3D viewing tools Neuroglancer (https://github.com/google/neuroglancer), webKnossos (https://github.com/scalableminds/webknossos), and CATMAID (https://github.com/catmaid/CATMAID).

## The cutting edge in vEM technology

The vEM field is currently expanding, with technologists and engineers delivering improvements in speed, throughput, and usability at pace (reviewed in ^1^). Sample slicing is becoming faster with the automation of ultramicrotomes and adoption of fast milling technologies from the materials sciences (plasma FIBs and gas cluster ion beams). Imaging throughput is increasing with tape feed-through systems (GridTape TEM) and multibeam SEMs that image the sample with multiple parallel electron beams (mSEM, FAST-EM). Automation of pipelines in image-acquisition software and monitoring systems aid both usability and error-free data collection over days, months and even years ^25^. So, while the technical capabilities are increasing fast, other issues such as accessibility and data handling and analysis are now major bottlenecks.

## vEM-associated technologies

Although vEM does not directly reveal the functional state of the cellular structures captured in the images, the integration of other spatial analysis technologies into correlative and multimodal workflows allows additional information to be mined from the sample. For example, X-ray microscopy of the entire sample volume adds context to smaller regions imaged by vEM, light microscopy adds dynamic molecular localisations (e.g. fluorescence microscopy), electron dense probes add static molecular localisations (e.g. APEX2, nanoparticles), nanoscale mass cytometry adds spatial chemical signatures (e.g. nanoSIMS, OribiSIMS, X-ray fluorescence), and spatial transcriptomics adds gene expression signatures.

*In situ* structural cell biology, where molecules are imaged in their cellular environment at near-atomic-resolution, is a related field that also depends on electron imaging. Although there are overlaps in some of the sample preparation techniques and microscopes, *in situ* structural cell biology is distinct from vEM because it is performed entirely at cryo temperatures and is limited to a continuous sample thickness of ~200 nm ^26^, which is too thin to be considered vEM by the current definition proposed by the community ^1^. There are, however, some nascent areas of crosstalk between the cryo and volume worlds, for example, the combination of cryo-fixation with vEM to improve sample preservation ^27^ and the use of cryo-FIB-SEM as a vEM imaging tool ^28,29^ (though both are limited to samples smaller than 200 microns in at least one dimension due to the physical limits of vitrification).

## Who are volume electron microscopists?

The demands of sample preparation and the expense and complexity of many of the vEM instruments and workflows have generally limited access to vEM to a small number of experts. There are many and varied paths to becoming a volume electron microscopist. For instance, to become a vEM expert in a core facility one may have studied biosciences; used light microscopy to image a sample; ran out of resolution or needed to see where the fluorescent protein was localised within the context of cell structure; acquired first EM images of a sample with the help of an EM expert; been captivated by the complexity and beauty of cell structure; started training in the full vEM workflow; joined a specialist EM lab or facility; and eventually become a vEM expert. In other settings, for example, in specialist connectomics and vEM technology development labs, multidisciplinary teams that efficiently integrate varied individual expertise are more common. Here, the early experiences and career paths of vEM experts are varied, involving fields such as engineering, physics, chemistry, data science and life sciences. A profile of a vEM expert may thus include the following traits: multidisciplinary (e.g. cell biology, light microscopy, X-ray microscopy, electron microscopy, big data, engineering); collaborative (tackling complex projects as part of a larger team); and patient (vEM projects take months or years, not days or weeks).

## Access to vEM?

Considering the limited number of vEM experts (currently perhaps a few hundred worldwide), the complex multimodal workflows, intricate sample preparation with toxic chemicals, and the expensive and sensitive instrumentation, vEM is well suited to the central facility access model. vEM microscopes have been gradually incorporated into existing EM facilities, but the diversity of vEM workflows means that there are few facilities large enough to offer all vEM options.

The choice of a particular vEM modality is driven by the scientific question, and incorporates considerations of sample size, target structure size, field of view and resolution required to capture the target structure, and availability of probes to highlight and confirm the identity of the target structure. vEM modalities overlap in their capability, and therefore one biological question might be addressed by e.g. ssTEM or SBF-SEM or array tomography, whereas another can only be answered by e.g. FIB-SEM. Thus, some biological questions may be answered by local EM facilities with access to only one or two vEM modalities, whereas others will require a large facility with the ability to flexibly combine multiple vEM (and perhaps other imaging modalities) into a custom workflow to produce a multiscale multimodal dataset that will answer the question.

The piecemeal incorporation of vEM into existing EM facilities and the low throughput of multiscale multimodal projects means that global vEM capacity is still low. When considering projected future vEM needs, the gap between supply and demand becomes more worrying, especially as the life sciences community becomes increasingly aware of the potential of vEM to reveal complex cell and tissue interactions across scales. We propose an urgent review of international supply and demand in volume EM, and construction of open access local, national and/ or international facilities to fill any gaps, alongside training programmes to ensure the availability of a sufficiently large skilled vEM workforce.

## The vEM community

The vEM community consists of imaging scientists who develop and deliver vEM, and end users who apply vEM to answer their research questions. The goal of the grass roots vEM community initiative is to build a coherent international community of vEM experts and users to facilitate science delivery, and to build capacity to meet the vEM needs of the bioscience research community.

## The international vEM community

In the 2000s, the ‘volume revolution’ gathered pace and so too did the growth of the vEM expert community. Despite local collaboration and networking by vEM experts, it was initially difficult to communicate the exciting advances being made in vEM to the wider life sciences community in the shadow of the burgeoning ‘resolution revolutions’ in both light microscopy and single-particle cryo-EM. Taking inspiration from the UK cryo-EM community, a vEM Town Hall meeting was organised by authors of this Comment (Collinson, Kleywegt, Patwardhan, Verkade), supported by and held at the Wellcome Trust in London (UK) in October 2019. Sixty members of the expert vEM community attended, representing biologists, microscopists, technologists, facility managers, hardware and software engineers, image analysts, public image archivists and commercial suppliers. Though there were international attendees, the meeting was driven by and focused on the UK vEM community, and from this meeting came the first formal proposal for a vEM infrastructure capable of meeting UK bioscience demand. The proposal suggested the formation of multiple centres of excellence for established vEM techniques (SBF-SEM, FIB-SEM, array tomography) housed close to key end-user communities in universities or research institutes, and a National Facility that would host newly developed vEM techniques that were not yet user-friendly enough for broader rollout (e.g. plasma FIB, multibeam SEM, GridTape TEM). All facilities would have the supporting hardware and expertise for sample preparation, correlative multimodal workflows (light microscopy, SEM, TEM, X-ray microscopy) and advanced data handling and analysis.

The proposal was submitted to UK funders who provided positive feedback but requested further development of the concept. Before further progress could be made, the COVID19 pandemic swept across the world. As with much of science, the vEM community continued their work online, holding Town Hall meetings in October 2020 (60 attendees) and October 2021 (200 attendees). In August 2020, at a vEM microlab workshop organised by another author of this Comment (Narayan), some 50 domain experts identified current issues in vEM, articulated as key ‘how can we?’ questions. These were: How can we… 1) Retrieve, preserve and prepare large volume biological samples to obtain appropriate information; 2) Accelerate end-to-end sample throughput and interpretation; 3) Improve software to transform vEM from a qualitative to a quantitative method, allowing user-friendly extraction of insights from biological samples; 4) Share vEM data quickly and efficiently for viewing and analysis without overburdening local storage and compute capacity; 5) Set up an efficient vEM facility; and finally, 6) Improve communication and information exchange within the vEM community and across disciplines?

**Figure 3:**
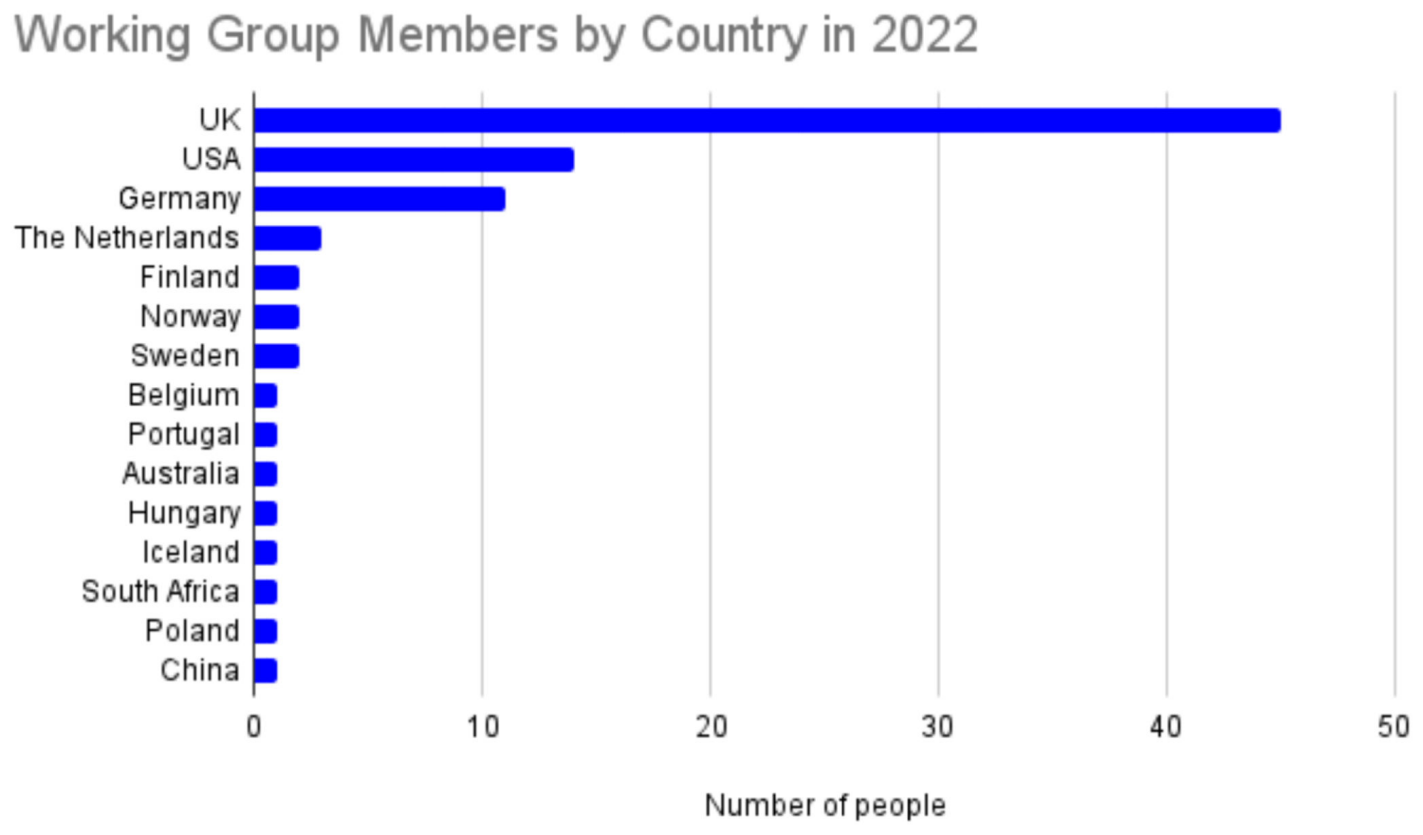
volume EM Community Working Group Membership by Country (2022).

From these meetings emerged two goals: 1) To build a coherent international community of vEM experts and users to facilitate science delivery; and 2) To build a vEM infrastructure capable of serving the needs of the international bioscience research community. To achieve these aims, six working groups were formed: Infrastructure, Community, Outreach, Sample Preparation, Data, and Training, each co-chaired by two volunteers from the vEM community (Table 1). As of early 2023, there are almost 80 international imaging scientists volunteering their time and enthusiasm across the working groups (Fig.3). As well as increasing the global representation of the working groups and networking colleagues across disciplines, the groups are delivering resources including:

- A new website to link the community and share resources (https://volumeem.org/)
- A mailing list for the vEM community (https://www.jiscmail.ac.uk/cgi-bin/webadmin?A0=VOLUME-EM).
- A vEM Twitter account (https://twitter.com/VolumeEM1) and hashtag (#volumeEM).
- A vEM community logo.
- Blog posts from the working group co-chairs on the community site FocalPlane (https://focalplane.biologists.com/category/blog-series/volume-em/).
- An edition of *Methods in Cell Biology* focused on vEM methods (Verkade P, Collinson L, Narayan K. Eds. (2023) Methods in Cell Biology, volume 177, Volume Electron Microscopy, Elsevier).
- A special issue of *Frontiers in Cell and Developmental Biology* focused on Correlative Light and volume EM: Methods and Applications (https://www.frontiersin.org/research-topics/22608/correlative-light-and-volume-electron-microscopy-methods-and-applications).
- A *Nature Reviews* Methods Primer on vEM for those new to the field ^1^.
- Case studies showing the impact of vEM in the biosciences (e.g. https://www.volumeem.org/case-studies.html).
- A new Gordon Research Conference focused on vEM starting in 2023 (https://www.grc.org/volume-electron-microscopy-conference/2023/).
- A list of volunteer vEM expert reviewers for journal editors and funders (available on request).
- A database of vEM-related resources setup in collaboration with Microlist (https://www.microlist.org/).
- A vEM-focused virtual seminar series in collaboration with Euro-BioImaging (https://www.eurobioimaging.eu/about-us/virtual-pub).
- A sample preparation widget in collaboration with the EMBL European Bioinformatics Institute (https://empiar.org/spw).
- Training videos to explain complex vEM workflows (https://www.volumeem.org/how-to-videos.html#/).
- Explainers of the toolkit required to get started with vEM workflows (https://www.volumeem.org/uploads/7/6/0/1/7601395/vemtoolkit.pdf).

## What next for vEM?

The impressive outputs of the grassroots vEM community have thus far been supported by the efforts of volunteers, all of whom have day jobs running imaging facilities and conducting bioscience research and technology development. The resources delivered to date demonstrate the power and the enthusiasm of the community for realising the full potential of vEM to drive discovery and translational research. However, delivering the next phase of community resources will require funding for dedicated people to coordinate and conduct larger scale activities. As of January 2023, we are happy to announce that the Chan Zuckerberg Initiative has awarded funding to the vEM community for this purpose through their ‘Advancing Imaging Through Collaborative Projects’ scheme (https://chanzuckerberg.com/science/programs-resources/imaging/community/?cycle=advancing-imaging-through-collaborative-projects).

Future resources planned by the community include:

- An annual vEM meeting for technologists to coordinate global efforts to further develop vEM into a quantitative imaging science (Community WG).
- A global map of vEM facilities to help bioscience researchers access expertise and instruments (Infrastructure and Community WGs).
- Development of a website for sharing expertise and spare capacity on instruments to make optimal use of existing vEM resources (Infrastructure WG).
- A series of training videos and animations explaining complex vEM workflows (Training WG).

Activities with a more technical focus that will support the development of community standards and FAIR data practices include:

- A project to gather and analyse sample preparation protocols from across the community, to help map the diversity of methods and identify critical steps, with an eye to improving reproducibility (Sample Prep WG).
- A project to create a vEM ontology to help standardise the metadata that describes vEM datasets (Data WG).
- A project that harmonises protocols and ontologies into a sample preparation widget implemented at EMBL-EBI, which will associate complex protocol metadata with every vEM image set stored in public archives, to support FAIR practices and metastudies of deposited data (Sample Prep and Data WGs).
- The creation of a collaborative computational project for vEM to support users and developers of vEM software. The vEM community is keen to follow community standards such as REMBI, the recommended metadata for biological images ^30^, and the EMPIAR team at EMBL-EBI is working with the Data working group to devise a REMBI-compliant data model that captures metadata from vEM experiments (Data WG).

In addition to supporting the community to self-organise and establish key standards and practices in vEM, the critical issue of capacity for conducting vEM experiments remains. Since the initial proposal was submitted to the UK funding bodies, we have gathered further supporting evidence for the demand for a vEM infrastructure. Based on discussions within and beyond the community, the proposal has evolved to incorporate *in situ* structural biology, encompassing correlative and multimodal imaging across scales, from proteins in cells to tissues and organisms. Building on a foundation of best possible structural and molecular preservation required for EM, this infrastructure would be unique in allowing researchers to understand the high-resolution spatial organisation of complex biological systems. Further development of the proposal will leverage the expertise of the growing community and incorporate globally relevant insights, since vEM working group members are based in six continents (North America, South America, Africa, Asia, Oceania, Europe). In building vEM capacity, we will critically consider equity, diversity, and inclusion, to ensure that the technology is open to all regardless of local conditions. As a relatively new community, we also have an opportunity to design and adopt new strategies for minimising the environmental impact of large-scale technical and data infrastructures into our programmes of work. By embedding these principles in our community and infrastructure, we will ensure that we are consistent in working towards improving the health of the population and the health of the planet.

If you have enjoyed this article and are interested in learning more, whether as a technical specialist or as a potential end user, we encourage you to make contact through our website (https://www.volumeem.org/), and to join this exciting, open, and welcoming community at the forefront of imaging science.

## Figures and Tables

**Table 1:** vEM Community Working Groups

Working Group	Remit	Co-chairs 2020-2022
Community	Support all those practically involved in vEM techniques, across sample preparation, ancillary techniques e.g. X-ray imaging, CLEM, and includes microscopists and data scientists.	Jemima Burden (University College London) Chris Peddie (Francis Crick Institute)
Outreach	Reach out to the wider international biomedical science community in the form of: 1) Education about the various vEM techniques 2) Engagement of stakeholders including end users, engineers, software developers, funders and the public.	Georgina Fletcher (BioImagingUK) Anwen Bullen (University College London)
Sample Prep	Curation of sample preparation resources for the vEM community, including a protocol library that encompasses preparation steps through to imaging, with a focus on discussion and dissemination of knowledge around why each step is done, health and safety information, and annotations to indicate options and when they might be useful.	Carles Bosch (Francis Crick Institute) Michele Darrow (Rosalind Franklin Institute)
Data	1) Investigate community requirements for data storage, transfer and archiving. 2) Investigate community requirements for software and data analysis platforms. 3) Promote the development and use of metadata ontologies. 4) Democratise access to data and tools for the whole community.	Kedar Narayan (Frederick National Laboratory, National Cancer Institute, NIH) Martyn Winn (STFC UKRI)
Training	Provide resources and training on both the technical skills and managerial skills relating to all aspects of vEM.	Errin Johnson (Oxford University) Raffaella Carzaniga (Francis Crick Institute)
Infrastructure	Define, seek funding for, build and run a coordinated accessible infrastructure for vEM to meet the needs of the UK bioscience research community and to facilitate similar applications elsewhere.	Cheng Cheng (ConnectomX) Charles Wood (Portsmouth University) Paul Verkade (Bristol University) Lucy Collinson (Francis Crick Institute)
